# MicroRNAs from peripheral blood mononuclear cells as biomarkers for detection of preclinical fibrosarcoma

**DOI:** 10.1186/1753-6561-7-S2-P2

**Published:** 2013-04-04

**Authors:** Adriane F Evangelista, Danilo J Xavier, Elza T Sakamoto-Hojo, Eduardo A Donadi, Geraldo A Passos, Marcia MCM Silveira

**Affiliations:** 1 Molecular Oncology Research Center, Barretos Cancer Hospital, CEP:14784-400, Barretos, SP, Brazil; 2 Molecular Immunogenetics Group, Department of Genetics, Faculty of Medicine, USP, Ribeirão Preto, SP, Brazil

## Background

Blood immune cells cooperate to prevent the progression of tumors through cancer immunosurveillance. Since activated peripheral immune cell clones trigger a sensitive transcriptional response upon recognition of tumors, which can be identified by transcriptional profiling, we hypothesised that peripheral blood mononuclear cells (PBMCs) could be used as reporters for cancer detection.

## Materials and methods

We used a model system in which groups of immunocompetent BALB-c mice were subcutaneously injected with different numbers of tumorigenic B61 fibrosarcoma cells. The groups of study were: (i) tumoral group with serial injections of 10^2^ to 10^6^ cells; (ii) negative control group represented by sterile nonpyrogenic saline, (iii) inflammation group by Zymozan (Sigma) and (iv) bacterial infection group by injection of 10^7^colony forming units [cfu] pool from mice feces. Mouse peripheral blood was collected three days after injection; blood samples (N=10) were pooled according to experimental conditions. Mononuclear cells were separated by centrifugation on a Ficoll-Hypaque cushion (GE Healthcare) and RNA was extracted using Trizol Reagent (Invitrogen). Samples were hybridizated on miRNA microarrays (Agilent).

## Results

We identified four microRNAs, miR-451, miR-144, miR-486 and miR-494, which were differentially expressed when compared to control groups, including inflammation and bacterial infection.

## Conclusions

Our results showed that PBMC microRNA expression profiling can serve as a sensitive method for detection of preclinical cancer.

## Financial support

FAPESP.

